# Biodegradable, bile salt microparticles for localized fat dissolution

**DOI:** 10.1126/sciadv.abd8019

**Published:** 2020-12-04

**Authors:** Hanieh Safari, Nicholas Kaczorowski, Michael L. Felder, Emma R. Brannon, Mita Varghese, Kanakadurga Singer, Omolola Eniola-Adefeso

**Affiliations:** 1 Department of Chemical Engineering, University of Michigan, Ann Arbor, MI 48109, USA.; 2 Department of Pediatrics, University of Michigan Medical School, Ann Arbor, MI 48109, USA.

## Abstract

Bile acids are proposed as therapeutic agents for various diseases, including liver diseases and obesity. However, oral or subcutaneous administration of a solubilized version of these drugs has limited efficacy and imposes unwanted side effects. Here, we describe a gold-templating method for fabricating stable, bile salt—cholate or deoxycholate—microparticles. The gold ions’ reduction at the oil-water interface in a double emulsion solvent evaporation process enables a gold–bile salt interaction and the formation of bile salt particles. We demonstrate that composite microparticles release cholate/deoxycholate into solution via a surface erosion process. We illustrate these particles’ capability to lyse adipocytes, both in vitro and in vivo, with minimal side effects, contrary to the Food and Drug Administration–approved salt solution that leads to severe inflammation and ulceration. Overall, particle-based cholate/deoxycholate opens opportunities for localized delivery of these salts, improving efficacy while minimizing side effects associated with oral and subcutaneous use.

## Teaser

We describe a novel gold-templating method for fabricating stable, biodegradable, bile salt microparticles with controlled geometry.

## INTRODUCTION

Bile salts are naturally occurring surfactants that help solubilize lipids in the small intestine and regulate several hepatic, biliary, and intestinal functions (*1*, *2*). Bile acids and their salts have been proposed as therapeutic agents for the treatment of different conditions, including bile synthesis and peroxisomal disorders (*3*, *4*), primary biliary cirrhosis (*5*), gallstones and bile duct stones (*1*, *6*), nonalcoholic fatty liver disease (*7*), type 2 diabetes (*7*), cancer (*8*, *9*), and for local removal of undesired fat (*10*). Two formulations of these salts are Food and Drug Administration (FDA) approved for human use, orally administered capsules of cholic acid for the treatment of bile synthesis disorders and liver dysfunctions (*3*) and subcutaneous injection of deoxycholic acid, Kybella, for removal of submental fat (*10*).

The activity of bile salts toward lysing of fat cells, in particular, has had the highest commercial value given the societal need. Body image disturbance and undesired fat deposits can be the underlying cause of various psychiatric disorders, e.g., body dysmorphic disorder and depression (*11*, *12*). Fat contouring treatments have been shown to lead to improved self-esteem and interpersonal relationships, emotional stability, and relief from depression and anxiety (*12*–*14*). Liposuction is currently the most commonly used approach for the removal of undesired fat deposits. However, the invasiveness, high cost, and associated long recovery time have limited patients from seeking this treatment (*15*). Therefore, substitute, noninvasive therapeutic approaches are required. In this regard, the subcutaneous injection of a bile salt solution, i.e., Kybella, is FDA approved, mainly for the removal of submental fat (*10*). However, the associated inflammation and bruising and the required multiple dosages have waned the excitement for the use of Kybella and limited its application to submental fat reduction (*10*).

Controlled or targeted drug delivery has long been used as an alternative approach for administering drugs to increase specificity while lowering side effects (*16*). Fabrication of particle-based, controlled-release systems composed of bile salts, therefore, can be a helpful strategy to address the identified limitations to the use of bile acids and their salts as therapeutics. However, the encapsulation of water-soluble salts within biodegradable polymer particles has proved challenging in the past due to the rapid diffusion of the cargo out of the carrier matrix during the fabrication (*17*). In this study, we describe a novel method for the fabrication of bile salt composite microparticles that demonstrate controlled-release properties. We used an in situ Au(III) ion reduction at the oil-water interface to enable the formation of stable, cholate/deoxycholate-based microparticles of various sizes and morphologies. We show that our composite bile salt particles kill adipocyte cells in vitro in a time- and concentration-dependent manner and destroy adipose tissue in vivo in genetically obese mice without inducing the observed inflammation and ulceration associated with the commercial salt solution. Last, bile salt particles were successfully loaded with rhodamine dye as a model drug, highlighting the potential for the delivery of additional agents alongside bile salts to enhance their therapeutic effect.

## RESULTS

### Fabrication of cholate-based composite microparticles

**Fig. 1:**
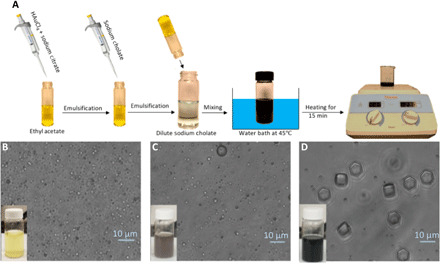
Illustration of the formation process of cholate-based composite microparticles via self-assembly at the oil-water interface. (**A**) Schematic illustration of the cholate-based microparticle fabrication process. The emulsion color and the shape of emulsion droplets as the heating and the reduction reaction proceed after (**B**) 0 min, (**C**) 10 min, and (**D**) 15 min of heating (scale bars of the bright-field images, 10 μm). Photo credit: H. Safari and M. L. Felder, University of Michigan.

In situ gold ions’ reduction within emulsion droplets has been used previously to fabricate gold-loaded, poly(lactic-*co*-glycolic acid) (PLGA) nanoparticles (*18*). In this work, we expanded this gold ion reduction process to the oil-water interface to enable the fabrication of bile salt composite microparticles via the well-established double emulsion solvent evaporation technique. Given its prior use as a surfactant in other systems, we first explored the fabrication of cholate-based particles (*18*). Figure 1A shows a step-by-step illustration of the process. The system’s inner water phase was doped with Au(III) ions and sodium citrate as precursors for gold nanoparticle formation. The well-known Turkevich method was used to reduce the gold precursor encapsulated within the emulsion droplets to form nanoparticles (*19*). The presence of an oil-water interface and gold ions’ reduction facilitated the assembly of cholate ions to yield hexagonal microparticles composed of 90 weight % (wt %) or more cholate in their structure. The system was then stirred to facilitate the evaporation of the organic solvent, and stable cholate particles were separated from the free gold nanoparticles via low-speed centrifugation. A scanning electron microscopy (SEM) image of cholate particles fabricated via this technique is shown in Fig. 1A.

During the heating stage, the gold ions’ reduction was signaled via a change in color of the reaction solution from yellow to dark blue–gray (Fig. 1, B to D). The self-assembly process and formation of the particles occurred after the completion of the heating step, i.e., Au(III) reduction, and before the stirring step, as shown in Fig. 1D. The presence of both the gold ion and sodium cholate was crucial for the formation of microparticles. When gold was eliminated from the process, no particles were formed. A minimum HAuCl_4_–to–sodium cholate mass ratio of 0.2 was also required to form microparticles. No cholate-based particle was formed below this limit, i.e., only gold nanoparticles were recovered from the sample. These observations demonstrate that particle formation is a direct consequence of the in situ Au(III) ion reduction and its interaction with the cholate salt. When the organic solvent was eliminated from the system and HAuCl_4_ was directly added to the sodium cholate solution, a white precipitate was immediately formed. Considering that cholic acid is a water-insoluble white powder and HAuCl_4_ is an acidic compound, the direct addition of these two compounds likely led to a substantial drop in the system’s pH, resulting in the formation of cholic acid powder. An organic solvent in the fabrication system prevented a pH drop, enabling the interaction of gold and cholate at the oil-water interface. This interfacial interaction of gold and cholate then facilitated the fabrication of cholate-based solid microparticles. The choice of solvent did not affect the self-assembly of the droplets because particles still formed with dichloromethane instead of ethyl acetate as the solvent in the system (fig. S1).

**Fig. 2:**
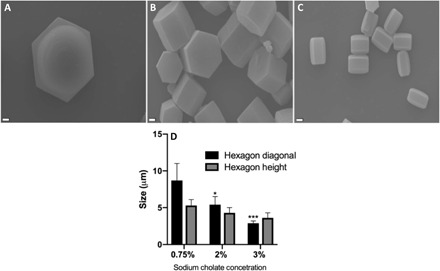
SEM image of the cholate-based hexagons with different sizes. Particles fabricated in the presence of (**A**) 0.75%, (**B**) 2%, and (**C**) 3% sodium cholate in the outer water phase. Scale bars, 1 μm. (**D**) Average size of particles as a function of sodium cholate concentration in the outer water phase; *n* = 3. Error bars represent SD. Significance is calculated with respect to the trial with 0.75% sodium cholate in the outer water phase via two-way analysis of variance (ANOVA) with Tukey’s posttest and confidence interval of 95%. *P < 0.05 and ***P < 0.001.

In line with previous studies (*20*), the size of the hexagons varied as a function of the surfactant concentration, as shown in Fig. 2 and fig. S2. As the surfactant concentration increased from 0.75 to 3%, the particle size decreased significantly from a diagonal of 9 μm down to 3 μm (Fig. 2D). Sodium cholate concentrations lower than 0.75% did not stabilize the emulsion and resulted in particles with unsmooth surface morphologies (fig. S3A). When sodium cholate concentrations of higher than 3% were used, particles with fibrous morphology were formed instead (fig. S3B).

### Characterization of composite microparticles

**Fig. 3:**
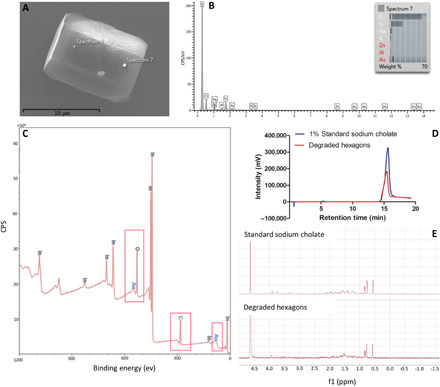
Chemical characterization of bile salt particles fabricated with the gold-assisted bulk templating method. (**A**) SEM image of the product of the fabrication process. (**B**) EDS analysis of cholate-based hexagons. The presence of silicon and other elements in the spectrum is the result of drying the sample on a glass slide. (**C**) X-ray photoelectron spectroscopy (XPS) analysis on the dried cholate hexagons mounted on indium foil, where CPS represents counts per second. Colored elemental labeling is added to the data, and pink boxes are drawn around the carbon, oxygen, and gold peaks to make the data more comprehensible. (**D**) High-performance liquid chromatography (HPLC) analysis on the degradation products of hexagons (red peak) and standard sodium cholate solution (purple peak) in a 50:50 mixture of acetonitrile and water. (**E**) Proton nuclear magnetic resonance (NMR) spectrum of 3% standard sodium cholate (top) and the degradation products of cholate-based hexagons (bottom) in deuterated water. The high-intensity peak showing up at 4.6 ppm is the solvent peak. The intensity of the peaks for degraded hexagons is increased using the MestReNova software to make the comparison between the two spectrums easier.

We used different techniques to characterize and confirm the presence of cholate in the microparticle structure. Energy-dispersive spectroscopy (EDS) was then used to identify the elements incorporated within the hexagon structure. Carbon was the main element present on the surface of the hexagons, as demonstrated in Fig. 3, A and B.

X-ray photoelectron spectroscopy (XPS) was used as a secondary method to confirm the elemental composition of the hexagonal microparticles. As shown in Fig. 3C, the sampled microparticles are composed of primarily carbon, oxygen, and gold—carbon and oxygen make up ~90 wt % with the rest being gold. Last, high-performance liquid chromatography (HPLC) and nuclear magnetic resonance spectroscopy (NMR) were used to confirm cholate’s presence in the structure of the microparticles. For HPLC analysis, the hexagons’ degradation product was run through an HPLC column alongside a standard 1.0% sodium cholate solution. As shown in Fig. 3D, the HPLC peak for the degraded hexagons (red peak) appeared at the same retention time as the standard sodium cholate (purple peak) solution. Figure 3E shows the proton NMR spectrums of the standard 3% sodium cholate solution and the degradation products of hexagons in deuterated water. Again, peaks for both the standard and particle degradation products were at the same chemical shift values, with the same splitting pattern in both spectrums.

### Fabrication of deoxycholate-based composite microparticles

Next, we explored whether our templating method can be modified to fabricate particles composed of other bile salts, particularly deoxycholic acid. Deoxycholic acid is a secondary bile acid formed by intestinal bacteria via the metabolism of cholic acid (*21*). As mentioned above, deoxycholic solutions have been used for local digestion of the fat tissue, with Kybella being its FDA-approved commercial formulation (*10*). Deoxycholate-based composite particles fabricated using the same protocol as described above, i.e., replacing sodium cholate with sodium deoxycholate, exhibit a rod-shaped morphology (fig. S4A). A concentration of 1% sodium deoxycholate in the outer water phase resulted in an average length of 8.4 ± 3.2 μm and a width of 870 ± 300 nm. The presence of the deoxycholate in the structure of the particles was confirmed via ^1^H NMR spectroscopy. The spectrum of the particle degradation products was then compared with the spectrum of a standard deoxycholate solution. As demonstrated in fig. S4B, all the standard deoxycholate solution peaks were present in the spectrum of the degraded particles, confirming deoxycholate as the main component in the structure of the particles.

### Characterization of the release profile and degradation kinetics of microparticles

**Fig. 4:**
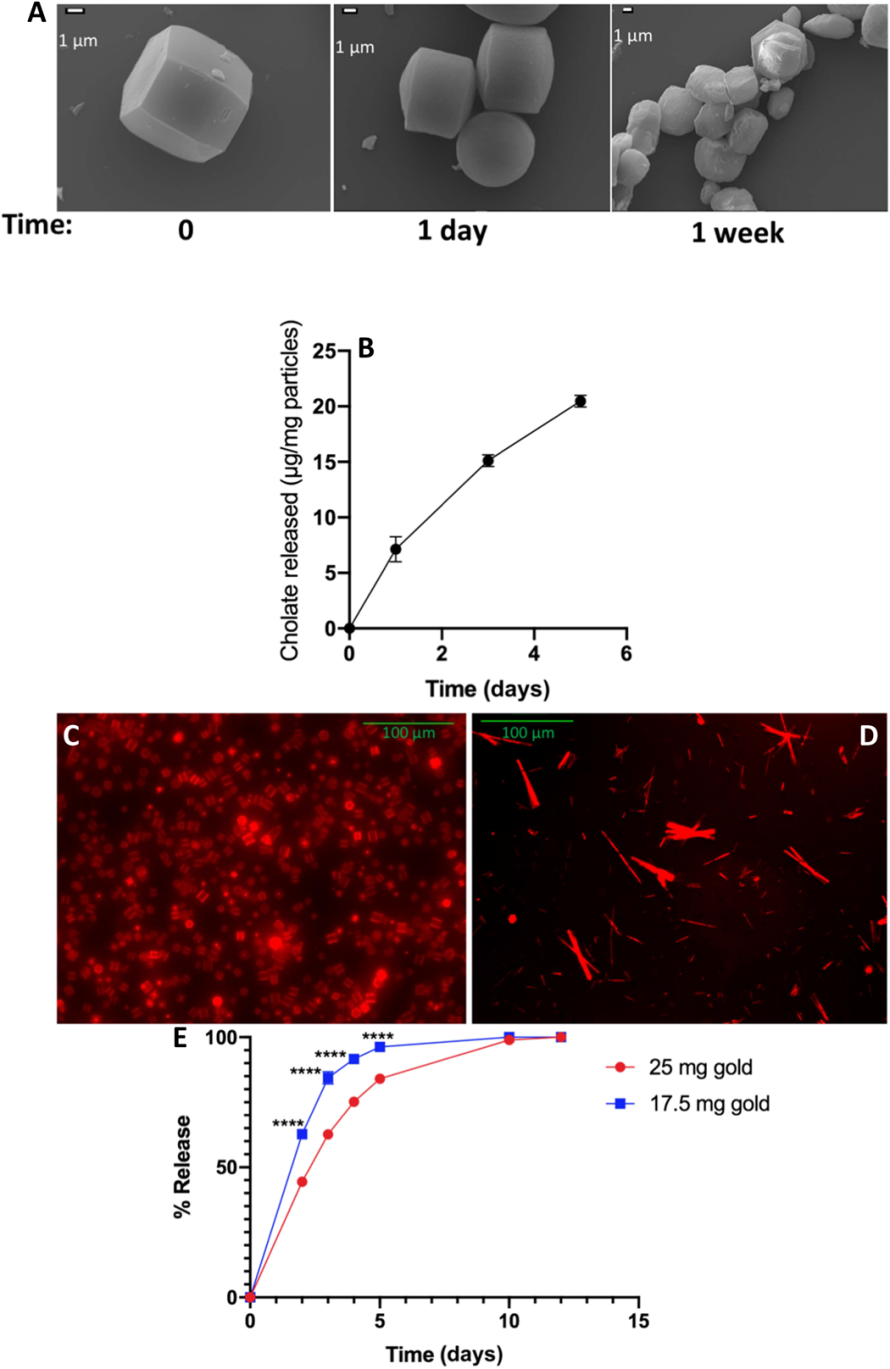
Representative degradation and release kinetics of bile salt microparticles. (**A**) SEM images of the surface morphology of cholate particles after incubation at 37°C for various time points. Scale bars, 1 μm. (**B**) Quantified amount of released cholate after incubation of bile salt particles at 37°C for different time points. Fluorescence image of rhodamine-loaded (**C**) cholate and (**D**) deoxycholate particles. (**E**) Release profile of rhodamine from deoxycholate composite particles fabricated in the presence of different amounts of HAuCl_4_. The concentration of the particles in phosphate-buffered saline (PBS) was set at 10 mg/ml. Each condition was repeated for *n* = 3 (error bars are smaller than 1% for all points). Two-way ANOVA was used to analyze (E), and significance for each point was calculated with respect to the corresponding condition fabricated in the presence of 25 mg of gold. ****P < 0.0001.

The cholate-based particles’ degradation kinetics was tested by incubating them in deionized water at 37°C and imaging them at different time intervals via SEM. A decrease in the overall size of incubated particles was visibly detected as early as 24 hours, which is due to erosion of salt from the particle surface. By 1 week of incubation, cracks were visible on the particle surface, with some particles broken into smaller sections (Fig. 4A). We speculate that surface erosion is the dominant mechanism for the degradation of the particles based on the visible intact internal structure of the particles throughout the degradation process. Quantification of the cholate released from degrading particles over time (concentration, 10 mg/ml) demonstrated a near-linear release profile, which is in line with the assertion that particle degradation is primarily by surface erosion (Fig. 4B) (*22*).

The absorption of a number of hydrophilic drugs can be enhanced with coadministration with or conjugation to bile salts, including cephalosporine, sotalol, and calcitonin (*23*). Thus, we demonstrated the production of cholate and deoxycholate microparticles loaded with rhodamine dye as a model hydrophilic agent (Fig. 4, C and D). When the loaded particles were subjected to degradation in phosphate-buffered saline (PBS) at 37°C, we observed a near-linear release profile of rhodamine up to day 5 (Fig. 4E). The observed release profile differs from the typical initial burst release of hydrophilic drugs from polymeric biodegradable particles where a diffusion-controlled release is dominant (*24*). Increasing the amount of gold ion in the precursor solution delayed the release profile of rhodamine from these particles, which we hypothesize is due to higher cross-linking in the particle matrix. The results demonstrate that we can tune the release of drugs from bile salt particles via adjustment of the amount of gold ion in the inner water phase.

### In vitro lysis of human subcutaneous adipocytes by bile salt composite microparticles

**Fig. 5:**
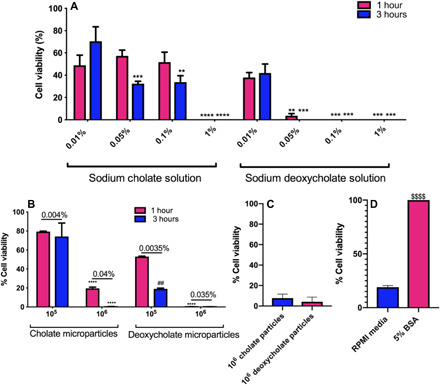
Representation of the death of primary subcutaneous adipocytes after incubation with bile salt particles. Cell viability of the primary subcutaneous adipocytes after being incubated with (**A**) different concentrations of the sodium deoxycholate and sodium cholate salts in RPMI media and (**B**) different concentrations of cholate-based and deoxycholate-based composite microparticles for different time points. Shown on top of the bars in each number concentration group are the correspondent mass concentrations for each condition calculated by counting the number of particles in a known mass. (**C**) Viability values for cells treated with supernatant from 10^6^ particles incubated in media for 3 hours (the degradation products of the particles). (**D**) Inhibitory effect of bovine serum albumin (BSA) on the lysis effect of deoxycholate-based microparticles, where 10^5^ particles suspended either in RPMI media or 5% BSA solution in RPMI media were added to each well. *n* = 3 for all assays. Cell viabilities were measured using MTS assay and calculated by dividing the net cell titer absorbance of the test wells by the untreated control wells. Two-way ANOVA with Tukey’s posttest and confidence interval of 95% were used to analyze (A) and (B), and unpaired *t* test was used to analyze (D). “*” represents the significance compared to 0.01% salt or 10^5^ particles per well of treatments, “#” compares the significance between 1-hour and 3-hour treatments, and “$” represents the difference between the treatment in 5% BSA versus RPMI media. No significant difference was observed between 1-hour and 3-hour treatments in (A). **P < 0.01, ***P < 0.001, and ****P < 0.0001.

Given the FDA approval of deoxycholate salt solution for the treatment of submental fat deposits, we tested the lipolytic capability of our bile salt particles, first in vitro via the use of commercially procured primary human subcutaneous adipocytes from individuals with a body mass index (BMI) >30. Adipocytes incubated with different concentrations of sodium cholate and sodium deoxycholate solutions in RPMI media served as positive controls. Consistent with previous studies (*25*), our results show both cholate and deoxycholate particles and their salt solution–induced cell death in cultured adipocytes, as demonstrated with the cell viability data reported in Fig. 5.

For the salt solutions, we found that sodium deoxycholate was more potent in disrupting fat cells than sodium cholate (Fig. 5A). Sodium deoxycholate solution concentrations higher than 0.05% were enough to kill fat cells within 1 hour, while a 1% or higher sodium cholate solution was necessary to achieve a 100% cell death. The difference between the cell viabilities was not significant when incubated with 0.1, 0.05, and 0.01% sodium cholate solutions (Fig. 5A). Similar to the salt solution assays, deoxycholate particles were more potent in killing the cells than the cholate particle. The addition of 10^5^ cholate particles in 150 μl of media per well, which would yield a salt solution concentration of 0.004% if particles were to degrade completely, minimally reduced cell viability by ~20%, independent of the incubation time. At a higher particle concentration of 10^6^ particles per well, the cell viability dropped to 20% by 1 hour of incubation, and to ~0% by 3 hours. For deoxycholate particles, 10^5^ particles per well (maximum effective soluble concentration of 0.0035%) were sufficient to decrease the cell viability to 60% within 1 hour of incubation and 20% after 3 hours. A deoxycholate particle concentration of 10^6^ per well was enough to kill all cells by 1 hour of incubation.

Next, we sought to demonstrate that the cell lysis in assays with particles was due to bile salt molecules released from degrading particles. We preincubated 10^6^ bile salt particles in 150 μl of media at 37°C for 3 hours. The particle solution was then centrifuged to remove undegraded particles and the supernatant, which contains released salts, added to fat cells for 3 hours. As shown in Fig. 5C, the particle supernatant successfully killed 90 to 95% of the cells within 3 hours, indicating that the degradation product of bile salt microparticles directly induces cell death in fat cells.

Previous studies have shown the inhibitory effect of bovine serum albumin (BSA) on bile salt–induced lysis of adipocytes (*25*). To confirm that our bile salt particles work with the same mechanism, we incubated 10^5^ of the deoxycholate particles with adipocytes in media or media with 5% BSA for 3 hours. Although particles led to cell death in media, this effect was inhibited entirely with the presence of 5% BSA in the solution (Fig. 5D). This observed result confirms that our composite bile salt particles lyse fat cells with the same mechanism as the salt, suggesting that our bile salt microparticles can be used as controlled-release systems for local digestion of fat tissue in humans.

### In vivo lysis of mouse adipose tissue by bile salt microparticles

**Fig. 6:**
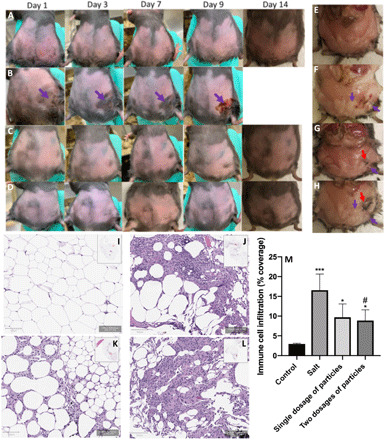
Investigation of the in vivo lipolytic performance of deoxycholate composite microparticles. The visual appearance of obese animals after receiving 100 μl of (**A**) pure saline, (**B**) sodium deoxycholate, and (**C**) one dosage and (**D**) two dosages of deoxycholate microparticles in saline at different times after injection. Salt (2.5 mg) or particles were injected into the right inguinal fat pad of animals in each trial. Purple arrows show the formation of an ulcer at the injection site in animals that had received the sodium deoxycholate injection. Posteuthanasia appearance of the inguinal fat pads of animals 2 weeks after receiving (**E**) pure saline, (**F**) sodium deoxycholate, and (**G**) one dosage and (**H**) two dosages of deoxycholate particles. Purple arrows show lipolysis sites in the right fat pad, and red arrows show the remains of particles at the injection site. Histology hematoxylin and eosin (H&E) sections of the adipose tissue for animals that had received (**I**) pure saline, (**J**) sodium deoxycholate, and (**K**) one dosage and (**L**) two dosages of deoxycholate microparticles. (**M**) Immune response in adipose tissue measured via extent of white cell infiltration into the tissue after receiving different treatments. Eight- to 10-week-old female B6.Cg-Lep^ob^/J animals were used for all the trials. Each trial was repeated for at least three different animals. One-way ANOVA with Tukey’s posttest was used to analyze (M). “*” and “#,” respectively, represent significance compared with the saline control and salt treatment. *P < 0.05 and ***P < 0.001. Photo credit: H. Safari and M. L. Felder, University of Michigan.

Assays to evaluate adipocyte cell death in vivo were performed via subcutaneous injection of rhodamine-loaded deoxycholate microparticles into the inguinal fat pads of genetically obese mice. Again, injection of the bile salt solution or saline in mice was used as the positive or vehicle control, respectively. Figure 6 presents a visual representation of the skin and fat tissue conditions at the injection site over time. Mice treatment with the vehicle control did not induce any local skin reaction (Fig. 6A). However, visible signs of severe bruising and inflammation were present at the injection site in the animals that received sodium deoxycholate solution, leading to the development of an ulcer 7 to 9 days after injection (Fig. 6B). These animals were euthanized immediately. There were no visual traces of skin inflammation or bruising in animals that received either one or two dosages of the deoxycholate particles, as shown in Fig. 6, C and D.

When animals were euthanized at 14 days after injection for particle and vehicle control treatments or immediately after ulcer formation for the salt treatment, visual evidence of local fat loss was observed in the animals that received both salt and particle injections. Clear sections of fat visible for the control (left) fat pad of animals were missing in the test (right) fat pad in the proximity of the injection site (Fig. 6, F to H). No such difference was observed between the right and the left fat pads of the animals that had received the vehicle control (Fig. 6E). The visual difference between the right and the left fat pads of animals was more prominent in the animals that had received two dosages of particles (Fig. 6H). In addition, particles were visible at the injection site 2 weeks after injection, confirming the gradual degradation of particles in vivo, which promoted lipolysis without severe inflammation. No significant change in the body weight of animals was observed during the lipolytic treatment for test or vehicle control groups (fig. S5), indicating a localized effect, as seen in the clinics with the salt formulation of deoxycholate (*26*).

It is known that the disruption of adipocyte membranes and solubilization of the fat tissue with deoxycholic acid trigger a local inflammatory response and the recruitment of macrophages to clear the cellular debris (*27*). Thus, immune cell infiltration in adipose histology sections is a significant indicator of the deoxycholic acid’s lipolytic performance. Therefore, we performed histological evaluations of excised fat pads from all the treatment conditions. In control animals with saline treatment, both right and left fat pad sections showed intact cells with no lysis (fig. S6 and Fig. 6I). However, significant cell lysis was depicted by fewer adipocytes in animals that had received bile salt treatments (Fig. 6, J to L). In hematoxylin and eosin (H&E) sections, leukocyte infiltration, characterized by crown-like structures (CLS), indicates inflammation and clearance of dead adipocyte cells (*28*). We observed CLS in the right fat pad of animals that received the salt or particle treatments (Fig. 6, K and L), but not in the saline control sections (Fig. 6I). Accordingly, quantification of the immune response in the adipose tissue samples demonstrated a significant increase in the percentage of the adipose histology sections covered with immune cell infiltrates in the pad of mice receiving either salt or particle treatments as compared with the pure saline injection (Fig. 6M). However, while the single dosage of particle treatment visually had lesser inflammation than the double dosage (Fig. 6, K and L), the percent coverage of immune cells was not significant between the two.

We also observed a decrease in the weight of the right (test) fat pad compared with the left (control) fat pad of animals after receiving either salt or particle treatments (fig. S7). However, the results were not significant from the saline treatment because of the fluctuations and inherent differences in the weight of the fat pads within each animal even before receiving any treatments.

Next, we sought to determine the therapeutic window for particles by identifying the maximum tolerated dosage capable of lysing adipocytes without skin reaction and ulceration. For this purpose, we injected particles into the right fat pad of animals at either two or five times higher dosage of the original treatment. Our results showed that after a two-time increase in the dosage of particles from 2.5 mg per animal to 5 mg per animal, 80% of animals (four out of five) did not show any skin reaction or ulceration (fig. S8A); one mouse developed a small ulcer 2 weeks after injection (fig. S8B). A five times increase in the dosage to 12.5 mg per animal led to an ulcer appearance in 40% (two of five) of animals (fig. S8C). Yet, no skin reaction was observed in the other three animals by the end of the study (fig. S8D). Spreading the five times (12.5 mg per animal) dosage of particles across the entire fat pad area at four different injection sites eliminated skin reactions and formation of any subsequent ulcer (fig. S8E).

Conversely, an evaluation of a five times lower salt solution dosage of 0.5 mg per animal, to see whether we can achieve a comparable adipocyte lysis level to particles without any skin reaction, still led to all animals showing bruising and skin irritation at the injection site (fig. S8F). In addition, 33% (one of three) of these animals developed ulcer at 2 weeks after injection (fig. S8G). These results show that deoxycholate particles can significantly reduce the side effects observed for the soluble salt formulation, even at 10 times higher dosage of particles than the salt solution (i.e., 5 mg per animal for particles compared with 0.5 mg per animal for the salt solution).

The results presented in this section show that local injection of the deoxycholate particles or salt solution led to fat cell lysis and some degree of inflammation. However, severe ulceration at the injection site reported as the side effect of sodium deoxycholate solution (i.e., Kybella) was absent with composite microparticles.

## DISCUSSION

Bile salts are used as therapeutics to treat many diseases, including bile synthesis and liver disorders, cancer, and the reduction in undesired fat (*4*, *8*, *9*). In this work, we developed a new metal-assisted bulk templating method to fabricate solid cholate and deoxycholate microparticles to control-release bile salts for therapeutic applications. The composite particles degraded via surface erosion and released bile salts into solutions in a near-linear fashion, making them suitable for use as a controlled-release system in applications where limited efficacy and side effects have restricted the usage of bile salts. Our in vitro and in vivo experiments demonstrated that bile salt microparticles induced cell death in adipocytes without ulceration of the skin at the injection site.

We hypothesize that a “nucleation and growth” mechanism governs the formation of bile salt microparticles. The gold ions’ reduction at the oil-water interface initiates the formation of composite microparticles. Low concentrations of cholate have been used as reducing and capping reagents to produce hexagonal or triangular gold nanoparticles in a single phase (*29*). In our system, gold nanoparticles self-assemble in the oil-water interface, serving as a template for the formation of the bile salt particles. First, there is the formation of gold nanoparticles within the emulsion’s inner water phase, which serves as a nucleus for the self-assembly of the bile salt shell around nanoparticles. The interaction between the hexagonal gold core and the carboxylate group of bile salt ions leads to the formation of the gold–bile salt microstructures. We were also able to fabricate both cholate and deoxycholate microparticles using this approach.

Two of the main therapeutic applications of bile salts include the use of orally administered cholic acid capsules (Cholbam) to treat bile synthesis disorders and subcutaneous deoxycholic acid injections (Kybella) for the reduction of submental fat (*10*). However, the required multiple dosages and side effects associated with these formulations have limited their use. For example, deoxycholic acid destroys the adipose tissue via the destruction of the cell membrane. However, the local injection of the drug can cause swelling, bruising, numbness, itching, skin tightness, nerve injury, and damage to the surrounding tissue (*30*). In addition, a series of three to five injection sessions at least 1 month apart is required to achieve the desired lipolytic effect (*30*). Thus, controlled-release systems composed of deoxycholate as substitutes for Kybella can help maintain a constant dosage of the drug at the target site, preventing the required redosing and a sudden increase in the local concentration of the therapeutic. The direct contact of particles with the target tissue in the fat lysis application can increase efficacy while minimizing the damage to the surrounding tissues. Our in vitro and in vivo results confirmed the lipolytic effect of our composite microparticles with no visible skin ulceration at the injection site, suggesting these particles would be suitable for clinical use. However, further evaluation of the tissue function, metabolism, and systemic lipids induced by the composite microparticles would be necessary for their translation to clinical use.

Our in vivo results also confirmed the presence of particles at the injection site 2 weeks after injection, which helps minimize the number of required dosages for the desired therapeutic benefit. The presence of deoxycholate particles at the injection site 2 weeks after injection can be attributed to the combined effect of their size and geometry. Phagocytosis by interstitial macrophages and draining into the lymphatic system are primary mechanisms for the clearance of subcutaneously injected particles (*31*). Particles with a size of larger than 150 nm will move very slowly from the injection site, and drainage into the lymphatic system can take days (*31*), which is even slower in obese subjects (*32*). Thus, phagocytosis by macrophages remains as the primary mechanism for the clearance of deoxycholate microrods. Previous studies have demonstrated that macrophages cannot successfully internalize elongated particles, and shape modulation is a useful strategy to inhibit the phagocytosis of microparticles by macrophages (*33*–*35*). As a result, the size and elongated shape of deoxycholate particles increase their residence time within the injection site. The fabrication method’s tunability allows control of the bile salt particle size and geometry toward improving their efficacy, thus minimizing or eliminating the need for redosing. Our future work will focus on this.

In summary, the results of this work will open avenues for bile salt particles to be used as controlled-release drugs for the treatment of obesity and different liver disorders, which we will evaluate more in-depth in our future in vivo work. However, in-depth evaluations would be required to characterize the maximum tolerable dosage of the particles in animals fully. For one, the systemic toxicity of the formulation via blood assays, e.g., liver function enzymes, needs to be evaluated before moving further in the study. In addition, given that immune cell recruitment after initial disruption of lipid membranes is a major contributor to the deoxycholic acid–induced clearance of fat tissue, it should be noted that there are significant differences in mouse and human immunology that could affect the clinical translation of results presented in this work (*36*). For example, micro/nanoparticles’ interaction with human and mice leukocytes can be very different from each other (*37*), and others have reported several physiological and metabolic differences between mouse and human fat depots (*38*). Thus, the formulation would need to be evaluated in larger animal models before moving to clinical studies. Last, the current system has not yet been successful in fabricating particles in the nanometer size range. While the micrometer scale of the particles presented in this work is beneficial for maintaining them in the fat tissue for an extended period, the system would need to be optimized for the generation of nanoparticles to enable their use for systemic therapeutic applications.

## MATERIALS AND METHODS

### Study design

In this study, we have used gold as a template for the fabrication of solid microparticles that are composed of bile salts. We then tested these particles alongside the commercial deoxycholate salt formulation and the vehicle control to assess their lipolytic capability both in vitro and in vivo. Each in vitro condition was replicated for three different cell wells, and the in vivo trials were replicated for three animals. Animals had the same age, and the average weight of animals in each group was matched before the initiation of the study.

### Study approvals

Animal studies were conducted following the National Institutes of Health *Guidelines for the Care and Use of Laboratory Animals* and approved by the Institutional Animal Care and Use Committee of the University of Michigan. Eight- to 10-week-old female genetically obese (B6.Cg-Lep^ob^/J) mice purchased from the Jackson laboratory were used in this study.

### Fabrication of bile salt–based composite microparticles

All the materials were purchased from Sigma-Aldrich. Microparticles were fabricated using the modified double emulsion solvent evaporation method combined with the in situ reduction of the Au(III) ions within the emulsion droplets. Briefly, sodium citrate (30 mg) and gold(III) chloride hydrate (25 mg) were dissolved in water (50 μl) and emulsified in ethyl acetate (1.0 ml) via vortexing for 15 s. Afterward, 2 ml of concentrated sodium cholate/deoxycholate solution (0.75 to 3%) was added to the first emulsion, and the mixture was vortexed for another 30 s. The emulsion was then added to 0.3% sodium cholate/deoxycholate solution (10 ml) and heated for 15 min at 45°C in a closed glass vial using a water bath. The emulsion was then stirred on a stir plate at 220 rpm at room temperature for 2 hours for evaporation of the ethyl acetate. Rhodamine-loaded particles were fabricated with the same protocol by adding rhodamine (2 mg) to the inner water phase. Large gold precipitates and larger particles were filtered out using a mesh filter with a pore size of 20 μm. Composite particles were then collected and separated from the gold nanoparticle via low-speed centrifugation at 600 rpm for 5 min and discarding the supernatant.

### Microparticle characterization

Microparticles were imaged with bright-field and SEM (JEOL JSM-7800FLV) microscopes. For SEM imaging, the samples were dried on a glass slide and coated with carbon and imaged with both secondary electron and backscattered probes. The size of particles was measured via FIJI software and reported as the average size of at least 50 particles from three different batches. EDS and XPS analyses were used to characterize the elemental composition of the hexagons. EDS analysis was performed on the microparticles using an Oxford XMaxN 80-mm^2^ silicon drift energy-dispersive x-ray spectrometer. For XPS analysis, the particles were mounted on indium foil, and the XPS analysis was done via a Kratos Axis Ultra XPS machine. HPLC and proton NMR analyses were used to confirm the presence of cholate as the primary component in the structure of the hexagons. For these analyses, dried particles were degraded in a 50:50 mixture of acetonitrile and water and centrifuged to separate the residual gold entrapped within their structure. The supernatant was collected and analyzed with HPLC alongside a 1% standard sodium cholate solution in 50:50 acetonitrile and water. The raw HPLC data of intensity for different retention times for both the samples were plotted using the GraphPad Prism software. For NMR analysis of the cholate-based particles, the degradation products were freeze dried with a Labconco lyophilizer and dissolved in deuterated water and analyzed with a Varian MR-400 NMR machine alongside a standard sodium cholate solution. For the deoxycholate-based particles, the spectra of the degradation products and a 1% standard solution were collected in a 50:50 mixture of deuterated acetonitrile and water.

### Degradation and release assays

For rhodamine release assays, dried particles were resuspended in 1× PBS in a concentration of 5 mg/ml and rotated on an end-to-end rotator at 37°C. At different time points, the suspension was spun down, and released rhodamine was quantified via fluorescence measurement using a plate reader and the respective excitation and emission wavelengths of 553 and 627 nm. Each condition was repeated for *n* = 3.

For the degradation studies, dried particles were resuspended in deionized water at the concentration of 10 mg/ml and incubated at 37°C on an end-to-end rotator. At different desired time points, a droplet of the particle suspension was taken and dried on a glass slide and imaged via SEM to visualize their surface morphology. The cholate release assays were performed via the same protocol. At the desired time points, the particle suspension was spun down, and the supernatant was collected. The amount of the released cholate was measured using a bile salt ELISA (enzyme-linked immunosorbent assay) kit purchased from Sigma-Aldrich (St. Louis, MO). The trials were repeated for *n* = 3.

### In vitro adipocyte lysis assays

The primary subcutaneous human adipocytes cultured in 96-well plates (BMI, >30) were purchased from Zen-Bio (Research Triangle, NC). Upon arrival, 150 μl of the media was removed from each well, and the cells were incubated at 37°C and 5% CO_2_. For the lysis assays, 150 μl of the salt solution or particle suspension of the known concentration in fetal bovine serum–free RPMI medium was added to each well and incubated with the cells for a known time point. The treatment solution was then aspirated, the cells were washed with warm PBS, and 150 μl of a 1:25 dilution in 1× PBS of MTS assay cell titer purchased from Promega Corp. (Madison, WI) was added to each well. Plates were incubated at 37°C and 5% CO_2_ for 3 hours, at which time the control wells appeared orange. The absorbance was measured at 490 nm. The percentage of the cell viability was quantified via subtracting the background cell titer absorbance from the absorbance of the desired point and dividing it by the average signal of the untreated cells after subtracting the background.

### In vivo adipocyte lysis assays

Genetically obese mice were anesthetized using isoflurane, shaved, and 100 μl of a suspension of rhodamine-loaded deoxycholate particles or the solution of deoxycholate salt in saline (25 mg/ml) was subcutaneously injected into their right inguinal fat pad. Pure saline (100 μl) was injected into the left fat pad of the animals as the control. The weight and appearance of the animals were tracked for 2 weeks. One group of the animals received a second dosage of particles on day 7. For toxicity assays, a range of dosages of deoxycholate particles in 200 μl of saline (2.5, 5.0, and 12.5 mg per animal) was injected into the right inguinal fat pad of animals in a single spot. A separate group of animals received 12.5 mg of particles in 400 μl of saline, with the dosage being spread across the entire area of the right inguinal fat pad and four different injection sites. To evaluate the toxicity of sodium deoxycholate solution at lower concentration ranges, 100 μl of the salt solution (5 mg/ml) in saline was injected into the right inguinal fat pad of the animals. Each condition was replicated for at least three individual animals. After 14 days, the animals were euthanized and the right and left fat pads of the animals were removed, weighed, and fixed in 10% formalin solution overnight. Histology slides of the samples were prepared via paraffin embedding and standard H&E staining. The histology slides were analyzed by physicians blinded to the study group, and the digital scans were analyzed using the QuPath software. For quantification of the immune response, we calculated the percentage coverage of adipose histology sections with immune cell infiltrates. Ten different snapshots of each sample were randomly taken, the percentage coverage in each snapshot was quantified using the FIJI software and manual threshold adjustment, and the result for each sample was reported as the average across all the snapshots. At least three individual slides were analyzed for each treatment, and the percent coverage was reported as average ± SD.

## Supplementary Material

http://advances.sciencemag.org/cgi/content/full/6/49/eabd8019/DC1

Adobe PDF - abd8019_SM.pdf

Biodegradable, bile salt microparticles for localized fat disolution

## SUPPLEMENTARY MATERIALS

Supplementary material for this article is available at http://advances.sciencemag.org/cgi/content/full/6/49/eabd8019/DC1

## Appendix

View/request a protocol for this paper from *Bio-protocol*.
