# Supplementary material for: Biodegradable, bile salt microparticles for localized fat dissolution
Source: Sci Adv. 2020 Dec 4;6(49):eabd8019. doi: 10.1126/sciadv.abd8019 (PMC7821899; doi:10.1126/sciadv.abd8019)
Supplement: http://advances.sciencemag.org/cgi/content/full/6/49/eabd8019/DC1 [file supp_6_49_eabd8019__index.html]

Science Advances | Science AdvancesAAASSearchScience AdvancesMenu

## Supplementary Materials

# Biodegradable, bile salt microparticles for localized fat dissolution

Hanieh Safari, Nicholas Kaczorowski, Michael L. Felder, Emma R. Brannon, Mita Varghese, Kanakadurga Singer, Omolola Eniola-Adefeso

Download Supplement

**This PDF file includes:**

- Figs. S1 to S8

**Files in this Data Supplement:**

- Adobe PDF - abd8019\_SM.pdf
