# Supplementary material for: Biodegradable, bile salt microparticles for localized fat dissolution
Source: Sci Adv. 2020 Dec 4;6(49):eabd8019. doi: 10.1126/sciadv.abd8019 (PMC7821899; doi:10.1126/sciadv.abd8019)
Supplement: http://advances.sciencemag.org/cgi/content/full/6/49/eabd8019/DC1 [file supp_6_49_eabd8019__1.pdf]

Science Advances
AAAS

advances.sciencemag.org/cgi/content/full/6/49/eabd8019/DC1

Supplementary Materials for

Biodegradable, bile salt microparticles for localized fat dissolution

Hanieh Safari, Nicholas Kaczorowski, Michael L. Felder, Emma R. Brannon, Mita Varghese,
Kanakadurga Singer, Omolola Eniola-Adefeso*

*Corresponding author. Email: lolaa@umich.edu

Published 4 December 2020, *Sci. Adv.* **6**, eabd8019 (2020)
DOI: 10.1126/sciadv.abd8019

This PDF file includes:

Figs. S1 to S8

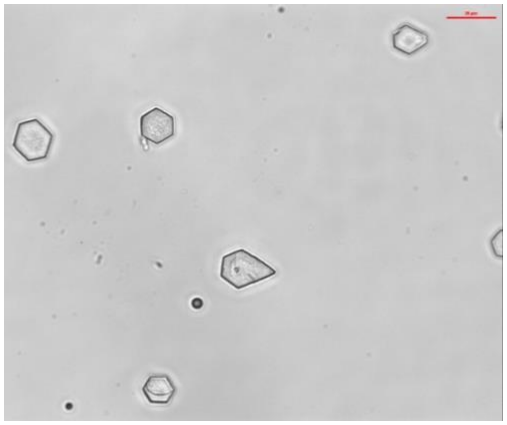

Fig. S1. The shape of the particles fabricated when dichloromethane was used as the solvent in the system. Scale bar is 20 µm in size.

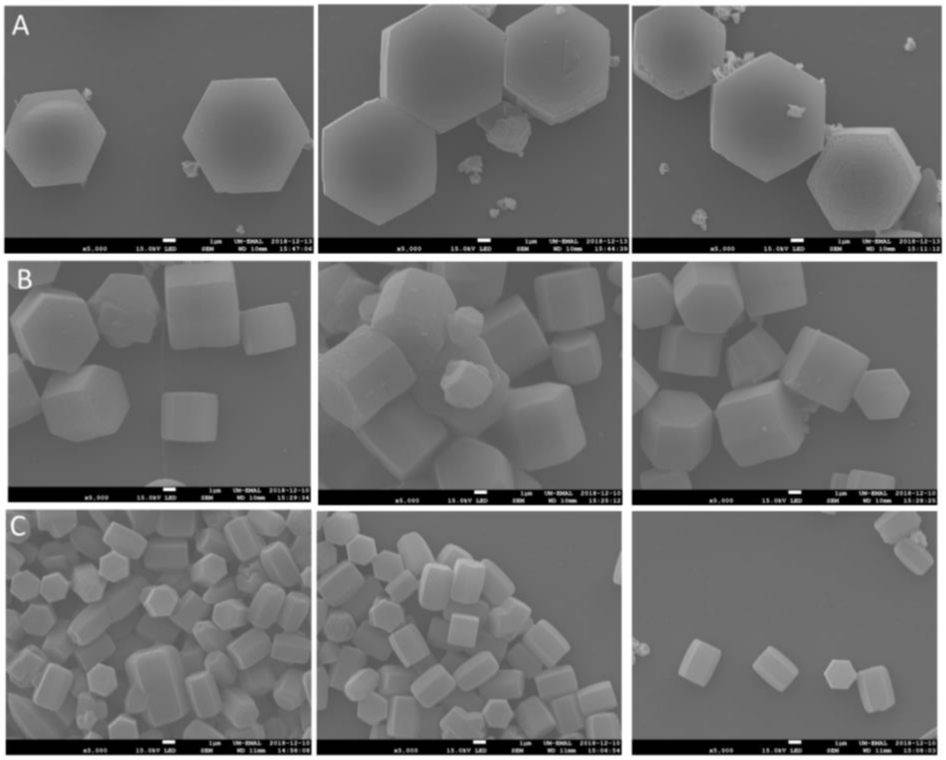

Fig. S2. **SEM image of cholate particles fabricated in presence of (A) 0.75% , (B) 2%, and (C) 3% sodium cholate in outer water phase. Scale bar = 1 micrometer in all images.**

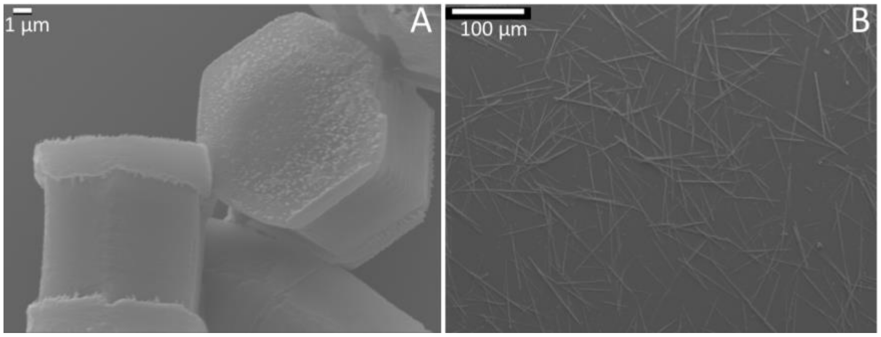

Fig. S3. **Surface morphology of cholate particles fabricated in a very high or very low concentrations of sodium cholate**. (**A**) SEM Image of the cholate-based hexagons with unsmooth surface fabricated in the presence of 0.5% sodium cholate in the outer water phase. (**B**) The image of the fibrous particles fabricated in the presence of 10% sodium cholate in the outer water phase.

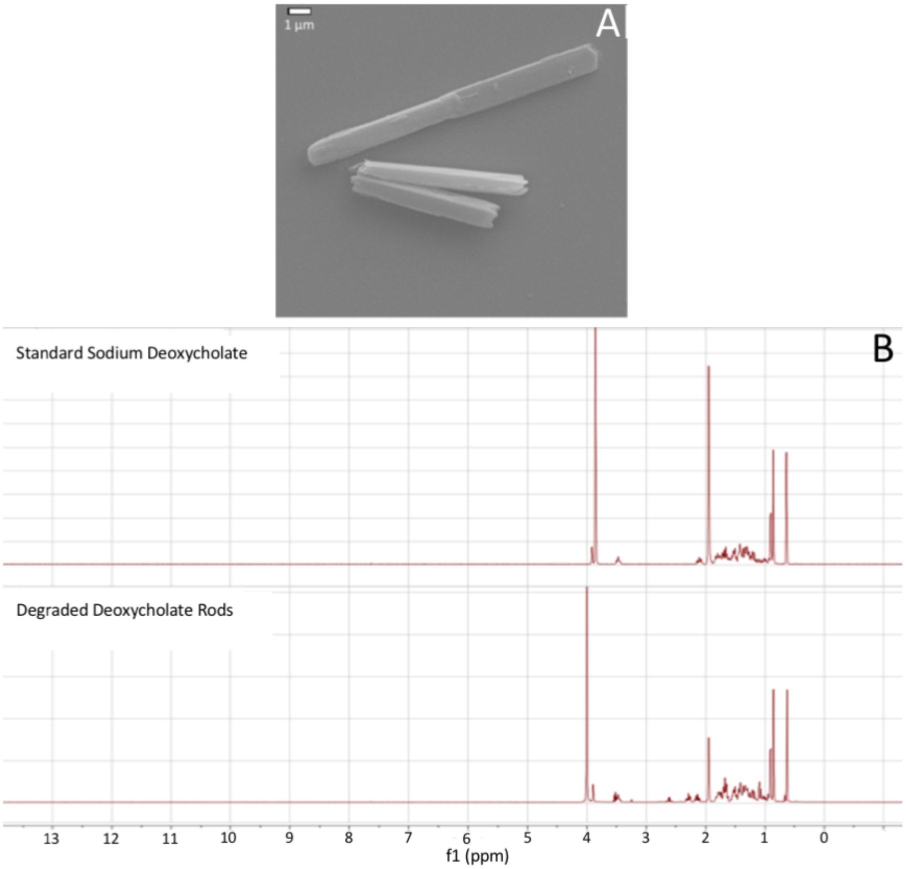

Fig. S4. Representation of deoxycholate-based microparticles fabricated via gold-assisted templating technique. (**A**) SEM image of deoxycholate-based composite microparticles. (**B**) Stacked NMR spectra of a standard 1% sodium deoxycholate solution and degradation products of the deoxycholate-based composite rods in a 50:50 mixture of deuterated acetonitrile and water. The two high intensity peaks showing up at the chemical shift values of 1.95 ppm and ~4 ppm are the solvent peaks.

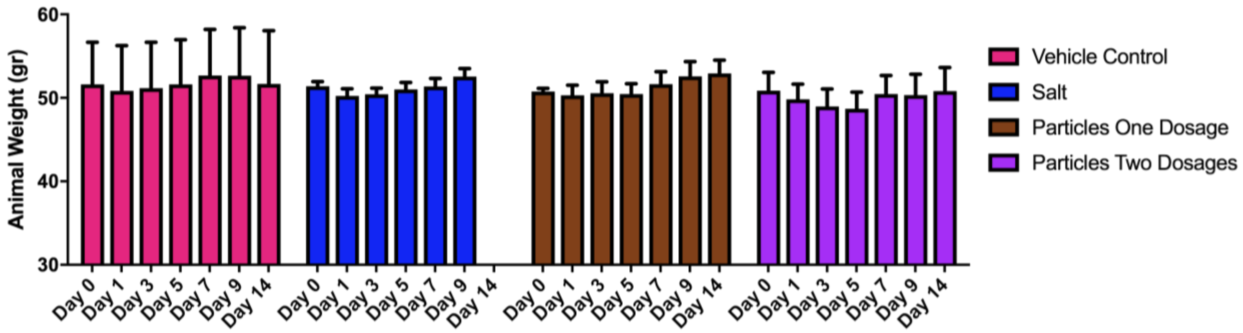

Fig. S5. **The weight of obese mice at different time points after receiving sodium deoxycholate salt or deoxycholate particles**. No significant difference in the weight of the animals was observed. Day 14 average weight for the salt is missing as that group of the animals had to euthanized at Day 9 due to development of an ulcer. 8-10 weeks old female B6.Cg-Lep^{ob}/J were used in all the studies.

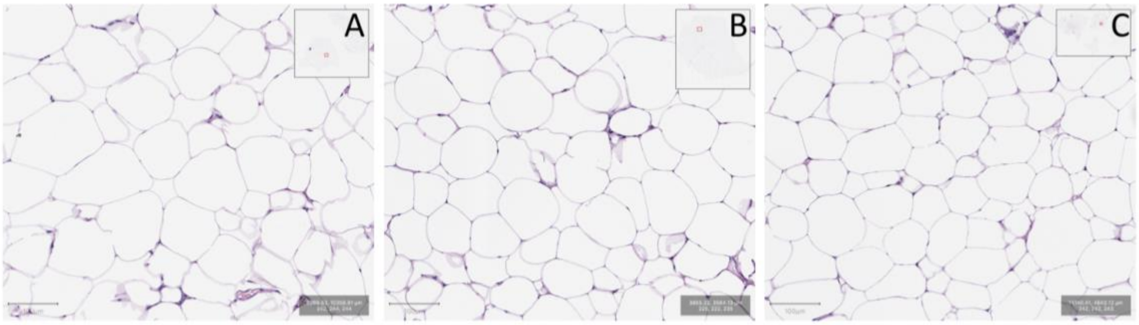

Fig. S6. **Histology sections of the left inguinal fat pad of obese animals that had received lipolytic treatment in their right fat pad.** The H&E histology sections of the left inguinal fat pad of the animals that had received (**A**) sodium deoxycholate, (**B**) one dosage and (**C**) two dosages of deoxycholate particles in their right inguinal fat pads. 100 μl of saline was injected into the

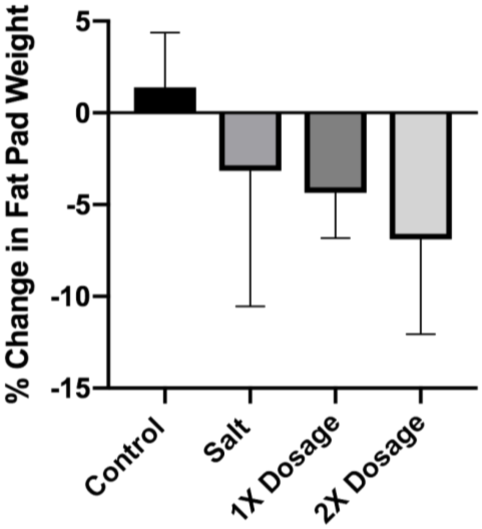

Fig. S7. **Quantified changes in the weight of inguinal fat pads of obese mice after receiving sodium deoxycholate salt or deoxycholate particles.** The percent change in the weight of the test (right) fat pad of the obese mice compared to their control (left) fat pad after receiving saline, sodium deoxycholate, and single or double dosage of deoxycholate particles. 8-10 week old female B6.Cg-Lep^{ob}/J animals were used for all the trials. Each condition was replicated for at least 3 different animals.

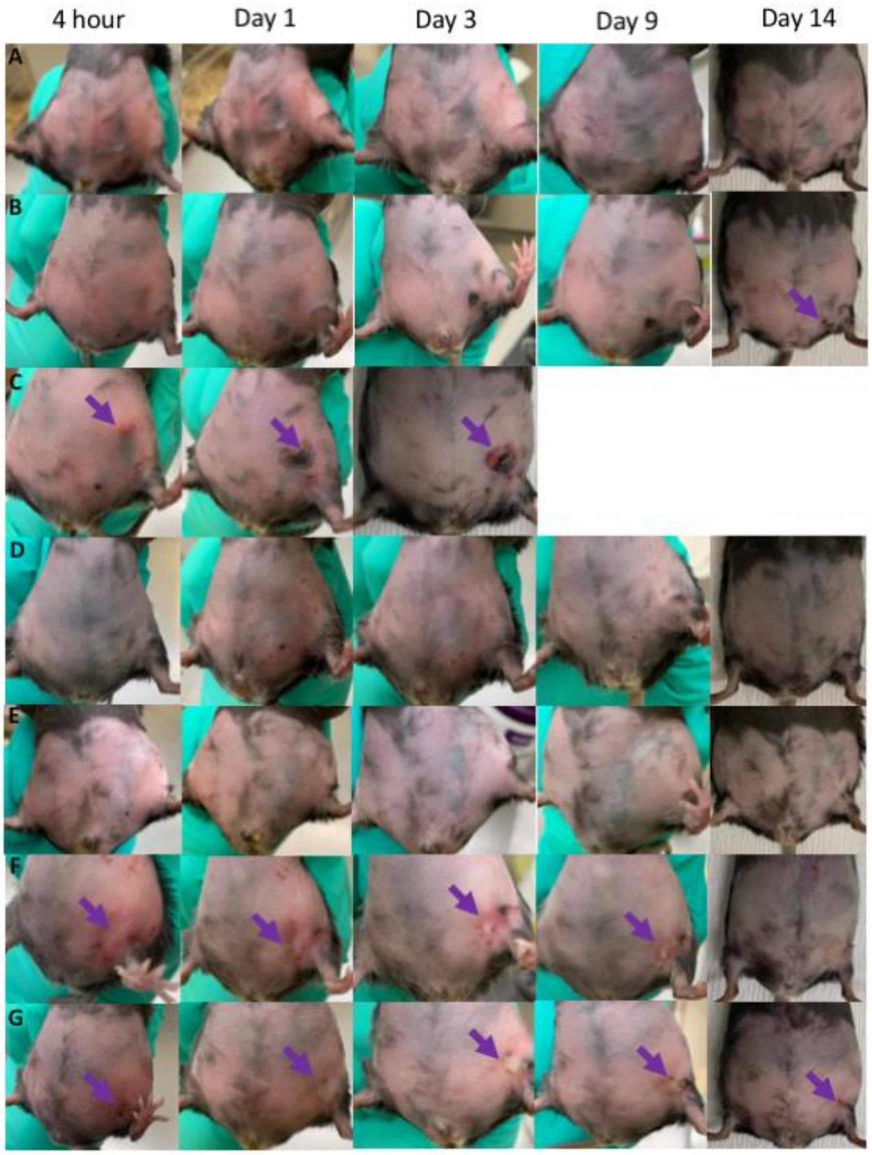

Fig. S8. **Cytotoxicity of different dosages of sodium deoxycholate and deoxycholate particles in obese animals.** The visual appearance of obese animals after subcutaneous injection of (**A, B**) 5 mg and (**C, D**) 12.5 mg deoxycholate particles in saline into a single spot in their right inguinal fat pad. (**E**) The visual appearance of the animals after receiving 12.5 mg deoxycholate particles with four injections spread across the area of their entire fat pad. (**F, G**) The visual appearance of animals after subcutaneous injection of the solution of 0.5 mg sodium deoxycholate in 100 µl saline. 8-10 week old female B6.Cg-Lep^{ob}/J animals were used for all the trials. Each trial was repeated for at least three individual animals. 20% of the animals that received 5 mg of deoxycholate particles and 40% of the animals that received 12.5 mg of deoxycholate particles via a single injection developed ulcers at the injection site while the other animals did not show any sign of skin irritation. Animals that received 12.5 mg deoxycholate particles via four injections spread across the entire fat pad area did not show any adverse skin reaction. All the animals that received 0.5 mg of sodium deoxycholate in the soluble form showed symptoms of skin irritation and bruising, and 30% of them ultimately developed ulcers. Photo Credit: Michael L. Felder, University of Michigan.
